# Transient Second Harmonic Generation Induced by Single Cycle THz pulses in Ba_0.8_Sr_0.2_TiO_3_/MgO

**DOI:** 10.1038/s41598-018-36686-5

**Published:** 2019-01-24

**Authors:** Kirill Grishunin, Vladislav Bilyk, Natalia Sherstyuk, Vladimir Mukhortov, Andrey Ovchinnikov, Oleg Chefonov, Mikhail Agranat, Elena Mishina, Alexey V. Kimel

**Affiliations:** 1 MIREA – Russian Technological University, Vernadsky Ave. 78, 119454 Moscow, Russia; 2 Southern Scientific Center of Russian Academy of Sciences, Chehova 41, Rostov-on-Don, 344006 Russia; 3 Joint Institute for High Temperatures of Russian Academy of Sciences (JIHT), Izhorskaya st. 13 Bd. 2, 125412 Moscow, Russia; 4 Radboud University, Institute for Molecules and Materials, 6525 AJ Nijmegen, The Netherlands

## Abstract

The ability to switch ferroics (magnets, ferroelectrics, multiferroics) between two stable bit states is the main principle of modern data storage technology. Due to many new ideas, originating from fundamental research during the last 50 years, this technology has developed in a breath-taking fashion. Ever increasing demands for faster and more energy efficient data storage strongly motivate fundamental studies of dynamics in ferroics triggered by ultrashort stimuli. It has been recently realized that nearly single cycle intense THz pulses and the phenomenon of the second harmonic generation are appealing tools for excitation and detection of poorly understood ultrafast dynamics of electric polarization in ferroelectrics at the picosecond timescale. Here we investigate picosecond dynamics of second harmonic from near-infrared pulse in ferroelectric heterostructure Ba_0.8_Sr_0.2_TiO_3_/MgO triggered by the electric field of a nearly single cycle intense THz pulse. The dynamics of the nonlinear optical signal is characterized by a step and oscillations at the frequency of about 1.67 THz. Although the observations can be mistakenly interpreted as oscillations of the electric polarization at the frequency of the soft mode and switching of the order parameter to another metastable state, here we show that the THz modulation of second harmonic generation in Ba_0.8_Sr_0.2_TiO_3_/MgO has a purely optical origin. The observation can be explained assuming that the THz pulse is a relativistically propagating inhomogeneity which induces center of symmetry breaking and linear birefringence. Our work reveals the role of propagation effects in interpretation of time-resolved non-linear optical experiments and thus it has important implications for experimental studies of ultrafast dynamics in ferroics.

## Introduction

Ultrafast magnetism, i.e. experimental studies of ultrafast spin dynamics triggered by femtosecond laser pulse, was pioneered 20 years ago^1^ being now a well-established research area^2^. In ferro-, ferri- and antiferromagnetic materials it has been demonstrated that femtosecond laser pulses can efficiently trigger coherent spin waves^2–5^, and even reverse magnetization. In particular, recently it was discovered that femtosecond laser excitation facilitates the fastest ever (20 ps) and least dissipative (20 aJ per 4000 nm^3^) write-read event in magnetic recording^6^.

Ultrafast ferroelectricity and optical control of spontaneous polarization in ferroelectric materials is far less understood. Electric fields are commonly used to manipulate the ferroelectric polarization, but the fastest ferroelectric recording takes at least hundreds of picoseconds^7^. The switching speed in these experiments has been mainly defined by the minimum duration of the electric field pulse that could be generated and applied to the medium via electrodes.

Possible scenarios to control electric polarization in ferroelectrics with the help of intense and ultrashort pulses of electromagnetic radiation have been analyzed theoretically^8,9^. It is believed that coherent control of phonons, and particularly phononic soft modes, can play in ultrafast manipulation of ferroelectricity a decisive role^10^.

Recent progress in generation of intense and nearly single cycle terahertz (THz) pulses^11–13^ opened the way for ultrafast and resonant excitation of coherent lattice vibrations. In particular, the electric field of such pulses can drive electric-dipole active vibrational modes by exerting forces directly on the charged nuclei.

The second harmonic generation (SHG) has long been one of the main techniques for studying the properties of the ferroelectric crystals and thin films^14–17^. Nonlinear optical measurements are sensitive to the local symmetry, crystallographic orientation, and polarization. Several groups reported about successful application of this technique as a probe of ultrafast laser induced dynamics in ferroics^18–24^. However, similarly to magneto-optics in ultrafast magnetism^3,25,26^, the problem of interpretation of the detected SHG transients has become one of the main obstacles in the experimental studies of ultrafast ferroelectricity.

Here, we analyze transient second harmonic generation induced by single cycle THz pulses in ferroelectric Ba_0.8_Sr_0.2_TiO_3_ film on MgO substrate. The observed SHG transients are as if the THz pulse triggered in the ferroelectric Ba_0.8_Sr_0.2_TiO_3_ coherent atomic oscillations at the frequency of the soft mode and caused a switching of the ferroelectric polarization. By analyzing the nonlinear optical response in time domain in experiments with co- and counter propagating pump (THz) and probe (near-infrared) pulses, we show that despite a good match of the observed frequency to the frequency of the soft mode in the compound, the observed oscillations have a different origin. The SHG transients must be interpreted taking into account effects of propagation^27,28^. In particular, the observed dynamics is due to interference of a static and a relativistically moving sources of second harmonic light.

## Results

We studied ferroelectric 400-nm thin film of barium strontium titanate (Ba_0.8_Sr_0.2_)TiO_3_. The film was fabricated by RF-sputtering of stoichiometric polycrystalline target on (111) MgO substrate (see Sample section for details). The X-ray diffraction analysis shows the high quality of the epitaxial growth and the orientation of the thin film respect to the substrate (see Supplementary Information for details).

**Figure 1:**
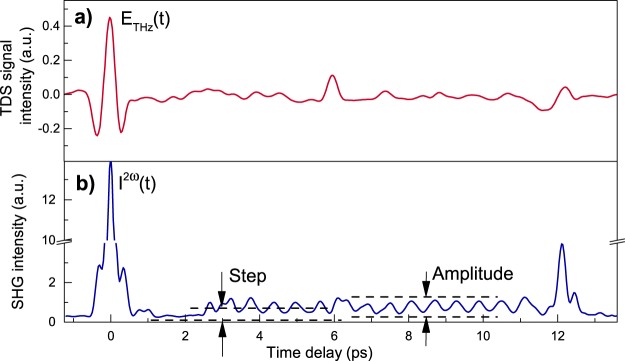
(**a**) The electric field waveform of the THz pulse transmitted through the sample and (**b**) dependence of the SHG intensity from Ba_0.8_Sr_0.2_TiO_3_/MgO on time delay for the case of co-propagating pump (THz) and probe (near infrared) pulses.

The electric field waveform of the THz pulse transmitted through the sample (Fig. 1a) was measured by THz time-domain spectroscopy technique (see Methods for details)^29^. Next to the strongest electric field at the delay of 0 ps, the traces of the nearly single cycle pulse are also seen at approximately 6 ps and 12 ps and correspond to roundtrip of the THz pulses due to reflection in the THz generation crystal OH1 (thickness, 440 μm; *n*_*THz*_ = 2.15)^30^ and in the sample (thickness, 580 μm; *n*_*THz*_ = 3.1)^31^, respectively.

The time dependence of the transient second harmonic generation (SHG) intensity is more complex (Fig. 1b, see Methods for details). During the action of the THz pulse on the Ba_0.8_Sr_0.2_TiO_3_ film, the nonlinear response follows the square of the THz electric field with the peak value of the SHG intensity about 140 times larger than the signal from the unexcited medium. Right after the action of the THz pulse, the intensity of the SHG signal is close to the signal from an unperturbed sample. Starting from 2.6 ps, the average level of the SHG signal goes up and starts to oscillate with a frequency about 1.67 THz around a constant non-zero level. The signal returns to the level corresponding to unperturbed medium after 12 ps. The frequency of the observed oscillations is very similar to the frequency of the soft mode in this material.

The effective time for the round-trip traveling of THz pulse corresponds to peak in transmission measurements and an abrupt quench of the oscillations in the SHG measurements. It means that the observed effects occur in time interval when THz pulse is present in the sample. Similar behavior was observed in the time-resolved measurements of the magneto-optical Faraday rotation^27^ that were explained in terms of propagation effects. To reveal the role of such effects, we carried out additional experiments with counter propagating THz and optical pulses^28^.

**Figure 2:**
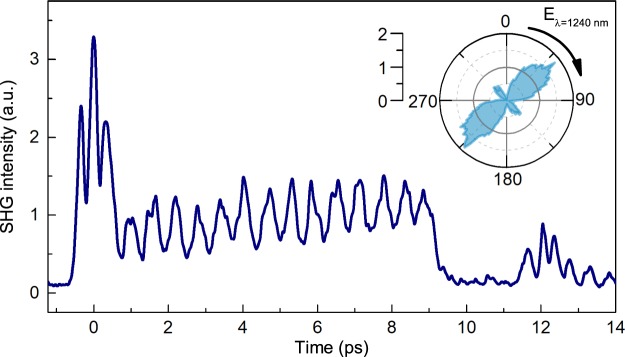
Dependence of SHG intensity on time delay for the case of counter-propagating pump (THz) and probe (near-infrared) pulses for the incident optical polarization – 0°; inset shows the dependence of the SHG intensity for Ba_0.8_Sr_0.2_TiO_3_/MgO on the orientation of the electric field direction of the probe pulse for non-exited state.

In case of counter-propagating geometry the Ba_0.8_Sr_0.2_TiO_3_ film was faced to the THz pulse while the substrate was faced to the optical pulse. The observed transient nonlinear-optical response in this geometry is shown in Fig. 2. There is no time lag and oscillations start right after the THz excitation. The frequency of the observed oscillations and the width of the step completely coincide with those observed in the experiment in the co-propagating geometry.

## Discussion

To describe the obtained results, it is necessary to consider all SHG sources and their corresponding contributions to the net nonlinear-optical signal.

If the THz pulse induces in the studied medium electric polarization P(E_THz_), it can be seen in the intensity of the second harmonic I^(2ω)^. For the SHG intensity in electric dipole approximation one finds that

1 $${{\rm{I}}}^{(2{\rm{\omega }})}\propto {|{{\rm{\chi }}}^{(2)}{{\rm{E}}}^{({\rm{\omega }})}{{\rm{E}}}^{({\rm{\omega }})}+{{\rm{\chi }}}^{(3)}{{\rm{P}}}_{0}{{\rm{E}}}^{({\rm{\omega }})}{{\rm{E}}}^{({\rm{\omega }})}+{{\rm{\chi }}}^{(3)}{\rm{P}}({{\rm{E}}}_{{\rm{THz}}}){{\rm{E}}}^{({\rm{\omega }})}{{\rm{E}}}^{({\rm{\omega }})}|}^{2},$$

where E^(ω)^ is the amplitude of the electric field of the infrared pulse, χ^(2)^ and χ^(3)^ are phenomenological tensors, P(E_THz_) is the electric polarization induced in the medium by the THz electric field E_THz_. The first χ^(2)^E^(ω)^E^(ω)^ and the second χ^(3)^P_0_E^(ω)^E^(ω)^ terms in this equation are meant to account for SHG from the ferroelectric medium. The second term is proportional to that part of the ferroelectric polarization P_0_, which is unaffected by the THz pulse. The first term does not depend on P_0_ at all. The third term accounts for the non-linear SHG source due to the electric polarization induced by the THz electric field P(E_THz_).

In electric-dipole approximation, SHG signal from unperturbed centrosymmetric MgO is zero (the first two terms in Eq. (1) are zero). External electric field of the THz pulse breaks the spatial inversion symmetry of MgO and results in the non-zero contribution to the net SHG signal (third term in the Eq. (1)). This is so-called THz Electric Field Induce Second Harmonic (TEFISH)^23,32^. For the non-centrosymmetric Ba_0.8_Sr_0.2_TiO_3_ film all terms in Eq. (1) are non-zero.

**Figure 3:**
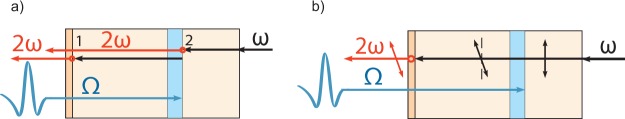
THz modulation of second harmonic generation by relativistic inhomogeneity for the case of counter-propagating near-infrared (probe) and THz (pump) pulses. Ω, ω and 2ω are schematically represent THz, infrared optical and optical second harmonic pulses, correspondently.

Note that for Ba_0.8_Sr_0.2_TiO_3_ TEFISH-term is an effect of higher order compared to the SHG sources represented by the first two terms. Consequently, for the ferroelectric film the first two terms dominate in the signal. In this case the origin of the step-like behavior of the transient SHG signal can be understood by taking into account the fact that a strong THz electric field induces an optical anisotropy in the isotropic MgO. It breaks the degeneracy between states of probe light polarized parallel and perpendicular to the electric field of the THz pump, respectively^33^. The propagating THz pump pulse in the MgO substrate acts on the probe pulse as a relativistically moving phase plate^34^. It results in the polarization rotation of the near-infrared probe pulse over angle, which depends only on the effective thickness of the THz-induced phase plate (Fig. 3b).

Since the Ba_0.8_Sr_0.2_TiO_3_ is an anisotropic material with the point group R3m^35^, the SHG intensity is extremely sensitive to the orientation of the electric field of the incident optical pulse (inset of Fig. 2). If the near-infrared probe pulse experiences a polarization rotation upon passing through the THz-induced phase plate and the SHG signal in this case will be different from the case of unperturbed medium. It results in a step-like behavior in ultrafast dynamics of the SHG signal.

The electric field of the propagating THz pulse breaks the space inversion symmetry (Fig. 3a) and induces a linear optical birefringence (Fig. 3b) in centrosymmetric and isotropic MgO. Figure 3(a) represents the case when second harmonic from the THz-induced relativistically moving region with the broken spatial inversion symmetry interferes with the SHG from the ferroelectric Ba_0.8_Sr_0.2_TiO_3_ film. Depending on the position of the region with the broken symmetry, the sources interfere constructively or destructively resulting in oscillations of the net second harmonic intensity. Figure 3(b) shows how the inhomogeneity changes the polarization of light resulting in a change of the net second harmonic intensity. Such changes results as a step in experimentally observed transient SHG signal.

In the case of counter-propagating geometry the pump and the probe pulses can still meet each other in the sample after the first overlap during $t=\frac{L}{c}({n}_{THz}+{n}_{\omega })$, where *n*_*THz*_ is refractive index for THz pulse, *L* is thickness of the MgO substrate. Taking into account that *n*_*ω*_ = 1.7188 and *n*_*THz*_ ≈ 3.09 at *ω* = 1 THz^31^, one finds that *t* ≈ 9.3 ps. This time agrees well with the time width of the experimentally observed step. It is important to note that this effect is observed only for counter-propagating pump and probe pulses. This fact clarifies the time lag (from 0.5 to 2.6 ps) in the experiment with co-propagating pulses (Fig. 1b). In this case, if THz and optical pulses co-propagate collinearly through the MgO substrate, they obviously have different group velocities. Time delay between infrared and terahertz pulses at the second surface of the substrate is ${\rm{\Delta }}t=\frac{L}{c}({n}_{THz}-{n}_{\omega })\approx 2.65$ ps. It means that after this time the infrared pulse will interact with the THz pulse reflected from the back surface of the MgO substrate and the step similar to the one seen in the counter-propagating geometry will emerge (Fig. 1b).

To explain the observed oscillations, we propose a model which accounts for interference of a static and a relativistically moving sources of second harmonic generation. Second harmonic can be efficiently generated by the Ba_0.8_Sr_0.2_TiO_3_ film. The electric field of the THz pulse also induces electric polarization in centrosymmetric MgO. The region with the THz-induced nonlinear optical polarization in MgO becomes an additional source of the second harmonic. The propagating THz pulse is in fact relativistically moving SHG source. The waves at the frequency 2*ω* from the static and moving sources will interfere such that the total intensity of the interfering waves will oscillate in time upon propagating of the moving SHG source (Fig. 3a)^32,36,37^. Depending on the time delay between the pulses, the sources will interfere constructively or destructively resulting in maxima or minima of the total SHG intensity. For instance, for each time delay ${\rm{\Delta }}t=\frac{{\lambda }_{2\omega }}{2c}{(1-\frac{{n}_{\omega }}{{n}_{2\omega }})}^{-1}$, the waves will interfere constructively and a maximum in the SHG signal will be observed. Considering the period of the observed oscillations Δ*t* ≈ 310 fs, the required ratio for the observed dynamic interference pattern is $|1-\frac{{n}_{\omega }}{{n}_{2\omega }}|\approx 3.3\cdot {10}^{-3}$.

Taking into account tabulated values of the refractive indices for MgO *n*_*ω*_ = 1.7188 and *n*_2*ω*_ = 1.7354^38^ at fundamental and doubled frequencies, we get $|1-\frac{{n}_{\omega }}{{n}_{2\omega }}|\approx 9.6\cdot {10}^{-3}\,$ that agrees with the experimentally estimated value with a good accuracy.

## Conclusions

Here we demonstrate the importance of effects of propagation in interpretation of results of nonlinear optical time-resolved experiments. Although second harmonic generation is established as a powerful probe of ferroelectricity, time-resolved measurements of ultrafast dynamics in ferroics with the help of this effect can be hampered by the presence of several interfering sources of the detected radiation. In particular, the dynamics triggered by nearly single cycle THz pulse in Ba_0.8_Sr_0.2_TiO_3_/MgO structure can be mistakenly assigned to the dynamics of the ferroelectric order parameter. Our experiments demonstrate that the observed nonlinear optical signal must be explained in terms of interference of the static and relativistically moving sources of the second harmonic in the studied structure.

## Methods

### Sample

We investigated a prototypical ferroelectric heteroepitaxial (Ba_0.8_Sr_0.2_)TiO_3_ [111] thin film (thickness 400 nm) on a centrosymmetric MgO substrate (thickness 580 μm). Ba_0.8_Sr_0.2_TiO_3_ film of such composition undergoes a ferroelectric phase transition at T_c_ = 353 K^35^. Moreover, Ba_0.8_Sr_0.2_TiO_3_ has several infrared active phonon modes in THz range that can be driven resonantly by THz radiation. One of these modes is the soft mode having the frequency Ω = 46 cm^−1^ (1.67 THz) at room temperature^39^. Magnesium oxide (MgO) is widely used as a substrate for thin films and heterostructures^40^. The material has a good transparency in the terahertz spectral range^41,42^ and thus suitable for THz lenses^43,44^.

### Experimental setup

A Cr-Forsterite laser system (center wavelength, 1240 nm; pulse duration, 100 fs; pulse energy, 65 mJ; repetition rate, 100 Hz) generates intense single-cycle terahertz pulses with the electric field amplitude up to 1 MV/cm and energy up to 7.5 μJ by optical rectification in organic crystal OH1.

To determine the electric field waveform of the transmitted through the sample terahertz radiation we used THz time-domain spectroscopy method. THz pulses transmitted through the sample and focused on a GaP crystal (thickness, 100 μm). These pulses induced a transient birefringence through the linear electro-optic effect. The birefringence and, therefore, the electric field were sampled step by step by a variably delayed infrared femtosecond laser pulse (central wavelength, 1240 nm).

For the nonlinear-optical measurements, the sample was placed in the focus of the THz pulse. The infrared probe optical pulse had co- or counter-propagation direction respect to the THz pulse. Upon non-linear interaction the THz pulse with the ferroelectric medium, second harmonic light was generated by the sample and was registered by a photomultiplier tube for different time delay between THz and optical pulses.

The schemes of the experimental setups are presented in Supplementary information.

## Electronic supplementary material

Supplementary
