# Supplementary material for: Transient Second Harmonic Generation Induced by Single Cycle THz pulses in Ba0.8Sr0.2TiO3/MgO
Source: Sci Rep. 2019 Jan 24;9:697. doi: 10.1038/s41598-018-36686-5 (PMC6346021; doi:10.1038/s41598-018-36686-5)
Supplement: Supplementary file 1 — Supplementary [file 41598_2018_36686_MOESM1_ESM.pdf]

TRANSIENT SECOND HARMONIC GENERATION INDUCED BY SINGLE CYCLE THZ PULSES IN $BA_{0.8}SR_{0.2}TIO_3$/MGO

KIRILL GRISHUNIN[1,4*], VLADISLAV BILYK[1], NATALIA SHERSTYUK[1], VLADIMIR MUKHORTOV[2], ANDREY OVCHINNIKOV[3], OLEG CHEFONOV[3], MIKHAIL AGRANAT[3], ELENA MISHINA[1], ALEXEY KIMEL[1,4]

[1]MIREA – Russian Technological University, Vernadsky Ave. 78, 119454, Moscow, Russia
[2]Southern Scientific Center of Russian Academy of Sciences, Chehova 41, Rostov-on-Don, 344006, Russia
[3]Joint Institute for High Temperatures of Russian Academy of Sciences (JIHT), Izhorskaya st. 13 Bd.2, 125412, Moscow, Russia
[4]Radboud University, Institute for Molecules and Materials, 6525 AJ, Nijmegen, The Netherlands
* K.Grishunin@science.ru.nl

Supplementary information

Sample characterization

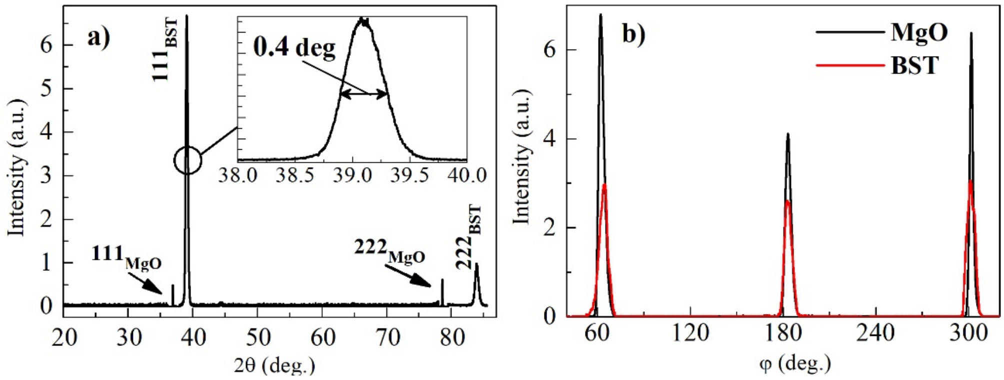

Fig.S1. X-ray diffraction scans: a) 2Θ–ω scan; inset - φ-scans of asymmetric 113 reflections; b) 2Θ–ω scan for BST film on (111) MgO substrate and pure MgO substrate.

X-ray diffraction (XRD) measurement of the $Ba_{0.8}Sr_{0.2}TiO_3$ film on MgO substrate reveals the absence of any additional impurity phase.

Fragment of the XRD pattern of the film is shown in Figure S1. The 2θ-ω XRD scan shows perfect orientation of the thin film along the [111] direction: the 2θ-ω XRD pattern reveals only HHH reflections, which confirms that [111] film || [111] MgO (Fig.S1a).

The quality of the epitaxial growth was confirmed by the φ-scans of 113 asymmetric reflections of the film and the substrate (Fig.S1b). The presence of three distinct components of the 113 reflection of film proves the full parallel epitaxial growth [111] film on [111] MgO.

Electro-optical sampling

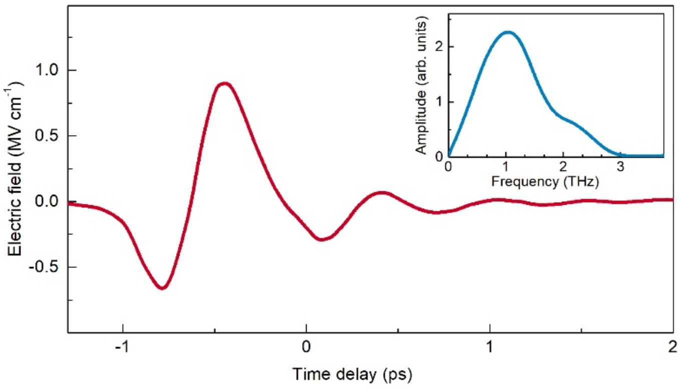

Fig S2. THz pulse profile and its frequency spectrum.

Experimental setup

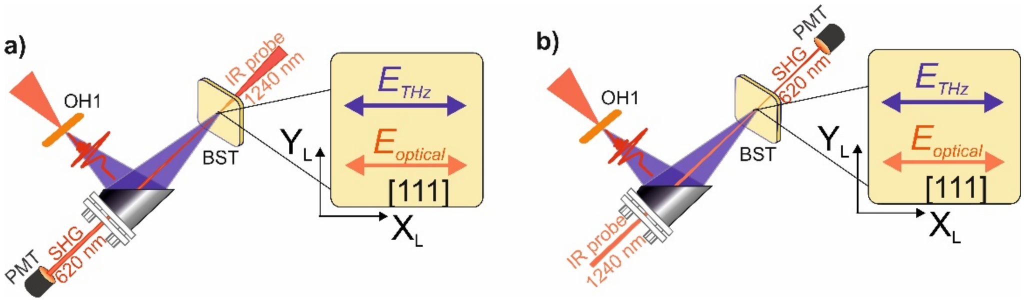

Fig S3. Experimental schemes of the THz-pump – nonlinear-optical probe for the a) counter- and b) co-propagating geometries.
